# The “Linked Health” of Diverse Caregivers: Insights from the National Social Life, Health, and Aging Project

**DOI:** 10.1093/geroni/igaf122.1645

**Published:** 2025-12-31

**Authors:** Lissette Piedra, Selena Zhong, Melissa Howe, Nell Compernolle

**Affiliations:** University of Illinois at Urbana-Champaign, Urbana, Illinois, United States; NORC, Chicago, Illinois, United States; NORC at the University of Chicago, Chicago, Illinois, United States; NORC at the University of Chicago, Chicago, Illinois, United States

## Abstract

This study explores the health of caregivers across five dimensions—physical, cognitive, mental, sexual, and sexual health—using the “linked health” concept, which suggests that changes in one health aspect affect others. We examine the correlation between caregivers’ ethnoracial and linguistic backgrounds and their overall health, with a focus on social networks. Data from 1,309 caregivers in Rounds 1-3 of the National Social Life, Health, and Aging Project were analyzed. Health was assessed through self-reports and clinical measures. The findings show that Black and Hispanic caregivers had poorer cognitive health and lower self-rated health compared to White caregivers, although functional health levels were similar. Larger social networks were linked to better cognitive outcomes. Black caregivers reported greater social strain and less support, while Spanish-speaking Hispanic caregivers experienced better social health and fewer mental health symptoms than English-speaking caregivers. These results underscore the need for targeted interventions to address health disparities among caregivers.

